# Migration experiences, employment status and psychological distress among Somali immigrants: a mixed-method international study

**DOI:** 10.1186/1471-2458-12-749

**Published:** 2012-09-07

**Authors:** Nasir Warfa, Sarah Curtis, Charles Watters, Ken Carswell, David Ingleby, Kamaldeep Bhui

**Affiliations:** 1 Centre for Psychiatry, Wolfson Institute of Preventive Medicine, Barts and The London School of Medicine & Dentistry, Queen Mary University of London, Charterhouse Square, London, E1M B6Q, UK; 2 Department of Geography, University of Durham, South Road, Durham, DH1 3LE, UK; 3 Rutgers University, Room 305, 405-7 Cooper Street, Camden, NJ 08102, USA; 4 Utrecht University, Heidelberglaan 2, 3584 CS, Utrecht, The Netherlands

## Abstract

**Background:**

The discourse about mental health problems among migrants and refugees tends to focus on adverse pre-migration experiences; there is less investigation of the environmental conditions in which refugee migrants live, and the contrasts between these situations in different countries. This cross-national study of two samples of Somali refugees living in London (UK) and Minneapolis, Minnesota, (USA) helps to fill a gap in the literature, and is unusual in being able to compare information collected in the same way in two cities in different countries.

**Methods:**

There were two parts to the study, focus groups to gather in-depth qualitative data and a survey of health status and quantifiable demographic and material factors. Three of the focus groups involved nineteen Somali professionals and five groups included twenty-eight lay Somalis who were living in London and Minneapolis. The quantitative survey was done with 189 Somali respondents, also living in London and Minneapolis. We used the MINI International Neuropsychiatric Interview (MINI) to assess ICD-10 and DSM-IV mental disorders.

**Results:**

The overall qualitative and quantitative results suggested that challenges to masculinity, thwarted aspirations, devalued refugee identity, unemployment, legal uncertainties and longer duration of stay in the host country account for poor psychological well-being and psychiatric disorders among this group.

**Conclusion:**

The use of a mixed-methods approach in this international study was essential since the quantitative and qualitative data provide different layers and depth of meaning and complement each other to provide a fuller picture of complex and multi-faceted life situations of refugees and asylum seekers. The comparison between the UK and US suggests that greater flexibility of access to labour markets for this refugee group might help to promote opportunities for better integration and mental well-being.

## Background

Many refugees have found new homes on the basis of the 1951 Geneva Refugee Convention [1]. Within this Convention, a ‘refugee’ is defined as:

“a person who owing to well-founded fear of being persecuted for reasons of race, religion, nationality, membership of a particular social group or political opinion, is outside the country of his nationality and is unable, or owing to such fear, is unwilling to avail himself of the protection of that country; or who, not having a nationality and being outside the country of his former habitual residence….is unable, or owing to such fear, is unwilling to return to it…”

An ‘asylum seeker’ is a person who has applied for asylum in a country other than their own and whose asylum application is still under consideration by the relevant authorities. An asylum seeker becomes a refugee if his or her claim for asylum is recognised by the host nation. Even though such laws offer legal protection of residency, they do not ensure optimal conditions of resettlement, and some would argue that the lengthy legal processes and the denial of employment rights for long periods are actually detrimental to health and wellbeing. Although there is limited epidemiological research on the mental health needs of Somali refugees living in different developed countries, single-country studies suggest a high level of mental health needs within refugee populations [2-4], attributing these to traumatic events prior to migration and the additional risk factors refugee groups face in the host nations [5-7].

Silove and his colleagues [7] provide a rich explanation of post-migration risk factors for psychological problems, particularly for populations exposed to mass trauma and displacement. The model comprises five systems: “personal safety”, “attachment and bond maintenance”, “identity and role-functioning”, “justice” and “existential meaning”. These are hypothesised to aid adaptation and survival and are threatened by war, human rights abuses and problems of acculturation. The model is inclusive in its approach and combines internal processes such as the psychobiological mechanisms underlying safety and attachment-bonding systems with wider individual, social and political aspects as recognised within the justice system, existential-meaning system and the identity/role system.

The model also provides a useful heuristic for understanding the development of psychological difficulties at the different stages of the refugee experience and the way in which interactions between different mechanisms may aid or hamper adaptation. It offers particular insight into the multiple influences on psychological well-being for refugees in host countries. For example, a delay in receiving refugee status may negatively affect the safety system through fear of return to the country of departure, the attachment-bonding system by preventing unification with family members and the identity/role system by preventing full engagement in society through employment or education. Similarly, negative attitudes in host country populations may affect the safety system, attachment-bonding system and identity/role system. The inter-relatedness of risk factors in the host country suggests that a particular risk factor may exacerbate other risk factors for poor mental health among refugee groups. Conversely, the receipt of refugee status and positive attitudes towards migrants may enhance feelings of safety and social support and increase social capital and the related systems.

Although the model allows for the consideration of the individual, social and political dimensions of the refugee experience, there is a limited understanding of how comparative social policies and geographical areas influence the genesis of mental illness and recovery among refugee populations living in different countries. Somalis are amongst the largest groups of refugees who have come to live in Europe and North America since the start of the Somali civil war. The Somali nation officially became a nation without a government in 1991, a stateless nation in which mass violence, bloodshed and human rights abuses were occurring on a daily basis [8]. Due to the collapse of the Somali state and the destruction of basic infrastructures, the Somali conflict has perpetuated human suffering in social, economic and political terms. Around 2 million Somalis have sought refuge in countries such as Kenya, Ethiopia and Yemen, while over 500,000 Somali refugees migrated to Europe, Australia, New Zealand and North America. This study aims to compare two samples of Somali refugees drawn from two urban settings in the UK (London) and USA (Minneapolis). The analyses investigate the similarities and differences in the perception of, and actual levels of, socio-economic and psychological problems among these two samples of Somali refugees.

## Research methods

The methods reported here were developed and tested in the Somali Mobility and Mental Health Study: SOMMER [9-12], a study of residential mobility and mental health in which the London data were collected. However, this paper provides an international comparison with new data collected from Somalis in Minneapolis in the US; these new data formed part of a follow-up PhD study. Ethical approval was obtained for the SOMMER study from Tower Hamlets and Lambeth Local Health Authorities; and for the PhD, from the University of Kent (Canterbury). Informed written or verbal consent was obtained from all the research participants**.** Further procedures were put in place to (a) identify those participants who might have been susceptible to psychological distress during the interviews and (b) inform these participants about the aims and intentions of the study, what was expected of them and what they could expect from the study. If a research participant became emotionally distressed, s/he was to be given the opportunity to terminate the interview. Vulnerable participants were also provided with leaflets containing information about specific voluntary or statutory health and social services that might be helpful or relevant to their needs, should they wish to take up such suggestions.

The SOMMER project investigated the prevalence and predictors of mental disorders among 143 Somali refugees living in two local boroughs of London [9]. It also explored and assessed the associations of pre- and post-migration geographical mobility and mental health problems of Somali refugees in East and South London [11,12]; as well as investigating the levels of met and unmet needs and health economic costs of mental illness in Somalis in London [10].

However, cross-national comparative studies can improve our understanding of which local factors are mediating or moderating influences on health status, as these conditions often vary in different countries. For example, asylum laws and restrictions on asylum seekers differ between the UK and USA. For this reason, we used the same research methods to recruit an additional interview data from a sample of 46 respondents and from three extra discussion groups of Somalis living in Minneapolis.

We wanted to know if Somali refugees living in the UK and USA face the same social and mental health problems, as well as examining if or how contextual and environmental differences between the UK and USA would have any effects on the levels of social and mental health problems of the two samples of Somali refugees living in these two countries. The group discussions were carried out before the quantitative data collection took place in London and Minneapolis. The following diagram shows the various stages of the data collection process between 2002 and 2005:

6 London qualitative focus groups (2001)

143 London quantitative Survey (2001–2003)

2 Minneapolis focus groups (2003–2004)

46 Minneapolis quantitative Survey (2004–2005).

### Focus group sampling

Given the lack of reliable, readily available population registers of Somali refugees, a convenience sampling method was used. This involved developing a map of locations from which Somali refugees could be recruited for focus groups and for the latter quantitative surveys. The procedures followed during the recruitment process are described elsewhere [9-12]. Briefly, these included: (a) researchers visiting Somali community organisations to explain the intention of the study and its expected outcomes; and (b) in view of the need to secure the trust of the community and thus successful recruitment, a comprehensive consultation process carried out in Somali shops, cafés and restaurants, mosques and money transmission (‘*Hawala’*) agencies. In this way, we developed an effective method of recruiting participants from areas of London and Minneapolis with significant numbers of Somali residents.

A total of 59 professional Somalis and lay Somalis between the age of 18 and 65 and from varied socio-economic backgrounds were contacted. The recommendation to hold separate discussions with the professional and lay Somalis was based on the advice of the two Somali mental health researchers. They pointed out that highly qualified Somali professionals tend to describe health problems using bio-medical terms, whereas Somalis with low educational qualifications or manual employment would more often refer to psychosomatic symptoms.

Of the 59 professional and lay Somalis invited to participate in the eight focus groups in London and Minneapolis, 47 participants attended. In Minneapolis, the lay and professional focus group discussions were held at Akmal Educational Centre, located within a popular Somali shopping centre (Karmel). In London, the focus group meetings were held at Queen Mary University of London, British Refugee Council and Somali community settings. We started the group discussions by asking the participants about their feelings and experiences when they first arrived in the host nations and if they had encountered any health and social problems since then. We asked the participants the following open-ended questions [11]: 

· Tell us your name?

· Where do you live?

· How did you feel when you first arrived in the UK/USA?

· In your experience, have you encountered any social problems since your arrival in the UK/USA?

· What kind of social problems do other Somalis experience?

· What do you think causes Somalis to have these problems?

· What health problems do you have, Somalis have?

· How do you/they sort them out?

· When do you think of mental health problem, what comes to your mind?

· What do you think could be the cause?

· Can you think of the impact of social problems on health?

· Is there anything else that we should have talked about but didn’t?

These open-ended questions were used to initiate conversation and guide the group discussions. The demographic and socioeconomic status of the discussion participants are presented in Tables 1 and 2 (see below).

**Table 1:** Demographic status of three professional focus group participants (age range: 25–55)

**Gender**	**Current occupation**	**City (Country)**
Female	Counsellor/Family Therapist	London (UK)
Male	Social Worker	London (UK)
Male	Housing Officer	London (UK)
Female	Housing Officer	London (UK)
Female	Advocacy Worker	London (UK)
Male	Somali Project Worker	London (UK)
Male	Counsellor/Caseworker	London (UK)
Female	Project Worker	London (UK)
Male	Psychiatrist	London (UK)
Male	Community Worker	London (UK)
Female	Case Worker	London (UK)
Male	Physician	London (UK)
Male	Housing Officer	London (UK)
Male	Immigration Advice Worker	Minneapolis (US)
Male	Community Representative (Office of the Senator)	Minneapolis (US)
Female	Social Worker	Minneapolis (US)
Male	Refugee Resettlement Programme Manager	Minneapolis (US)
Male	Human Resources Representative	Minneapolis (US)
Male	Employment Counsellor	Minneapolis (US)

**Table 2:** Demographic status of five lay focus group participants (age range: 19-65)

**Gender**	**Current occupation**	**City (Country)**
Female	Housewife	London (UK)
Female	Unemployed	London (UK)
Female	Unemployed	London (UK)
Female	Unemployed	London (UK)
Female	Housewife	London (UK)
Female	Pensioner	London (UK)
Female	Unemployed	London (UK)
Male	College Student	London (UK)
Male	Unemployed	London (UK)
Male	Asylum Seeker	London (UK)
Male	University Student	London (UK)
Female	College Student	London (UK)
Female	Housewife	London (UK)
Female	Housewife	London (UK)
Female	Unemployed	London (UK)
Male	Unemployed	London (UK)
Male	Unemployed	London (UK)
Male	Unemployed	London (UK)
Male	Asylum seeker	London (UK)
Female	College Student	Minneapolis (US)
Male	Taxi Driver	Minneapolis (US)
Female	University Student	Minneapolis (US)
Male	Sales Assistant	Minneapolis (US)
Male	Taxi Driver/Student	Minneapolis (US)
Female	Occupation not stated (works part-time)	Minneapolis (US)
Male	Security Guard	Minneapolis (US)

### Qualitative data analysis

Systematic manual text analyses of the focus groups were performed separately by using the Framework Analysis method [13]. The framework method consists of a number of stages including familiarization with the transcribed interviews, identifying a thematic framework, indexing key themes and sub-themes, charting, mapping and interpretation. This process of thematic identification was repeated using Nvivo [13], a qualitative software package, to enhance the reliability of coding. The views, experiences and themes of each group were recorded, transcribed, extracted and then examined in charts separately, before being contrasted across individuals and groups.

### Quantitative survey design (sampling & methodological challenges)

Carrying out epidemiological research with transient populations such as refugees and asylum seekers is challenging, not least because a representative sample is almost impossible to obtain through conventional random sampling methods [4]. The resulting shortage of empirical data makes it hard for social and healthcare professionals to consider appropriate public health interventions for vulnerable populations who are affected by numerous social problems and mental illness [14,15]. These populations include asylum seekers and refugees, homeless people, groups with substance misuse and mental illness, and irregular or undocumented migrants [16,17]. Data are not available on the exact number of Somali refugees in Britain and America, nor was it possible to determine from routine administrative sources the number of Somalis who were living in the locations where we recruited participants. Since there are no other international studies of mental health in Somali refugees that provide larger samples or which have a comparative study design, we spent considerable time and resources on identifying and recruiting from the geographical concentration of Somali refugees in certain parts of London and Minneapolis.

We used the aforementioned community engagement and triangulation approaches to engage Somali refugees in the study. Two Somali researchers spent considerable time (including weekends and evenings) in local community venues and talking to local Somalis in their usual social networks. This active engagement of the Somali community in the research process increased the levels of trust between the researchers and the community, minimised the amount of suspicion from potential for our later quantitative study, and enhanced the overall level of knowledge capture. The community engagement and focus group strategies we developed during the early stages of the study allowed us to have access to a group of hard-to-reach participants from conventional sites (Health Centres) and non-conventional sites such as Somali cafes, malls, shops and *Hawala* agencies [9,11]. We recruited and interviewed 189 Somali respondents between the age of 18 and 65. Seventy-six percent (143) of the respondents were from London and the remaining forty-six (46) from Minneapolis.

### Outcome measures

We used a number of research instruments. Of these, the demographic questionnaire and the Mini-International Neuropsychiatric Interview (MINI) are particularly relevant to this paper. The demographic questionnaire asked information on gender, date and place of birth, area of origin, marital status, history of family separation, religion, educational background, past and current employment experiences and immigration status. The MINI was used to measure major depression, post-traumatic stress disorder (PTSD), suicide, dysthmyia, current panic no agoraphobia, current panic and agoraphobia, obsessive compulsory disorder, current substance dependency, current substance abuse, current and life time psychotic disorder, and generalised anxiety disorder [18,19].

### Translation and cultural adaptation of MINI

The MINI has been used in 42 other languages and has good validity and reliability measures. It is also a highly structured instrument that is suitable for lay and non-clinical interviewers with the relevant training. The MINI interview produces ICD-10 and DSM-IV indicators of compatible psychiatric outcomes and is widely used in measuring mental health problems of clinical and normal populations [19]. The validity of psychiatric questionnaires designed for use in cross-cultural settings will depend upon an adequate translation process. The process of translating any document from one language into another is always complex because of linguistic and cultural variations. Most cross-cultural researchers in the field of social psychiatry are now familiar with the term ‘category fallacy’. Kleinman[20] proposed that: “In a local world of culture, interpretations are always judgments whose reliability may be determined by consistency of measurements but whose validity needs to be established through understanding that particular cultural context” (Kleinman, 1987: 447–454).

Since MINI has not previously been used or validated for use among Somali refugees and because of the substantial conceptual, cultural, contextual and linguistic differences between the English and Somali languages, we used Flaherty’s [21] five main dimensions of cross-cultural equivalence in validating and translating research instruments and thus avoiding categorical fallacy. To maintain the validity and reliability of the original instrument, a scientific steering group was set up to guide the translation process [9].The steering group consisted of three Somali PhD bilingual researchers, two bilingual Somali medical doctors, a bilingual Somali psychologist, two professors of social and community psychiatry, a professor of health geography and a professor in health economics.

Warfa [22,23] gives a detailed account of how Somalis construct and conceptualize mental illnesses through cultural symptoms and culturally perceived deviant behaviours. Somalis do not distinguish various types of mental disorders, for example, depression, dysthymia, general anxiety, stress, distress, mood swings and psychotic disorders. Nevertheless, Somalis have been using an elaborate local diagnostic system, similar to the DSM, to identify the traditional pathways to mental illness. For example, *wuu niyad jabay* (he or she dejected); *walwal badan ayaa haya* (he/she worries excessively); *wey murugeesantahay* (she is sad); *Jin baa galay* (Jin possessed him/her); *wey isla hadlay* (She started to talk to herself); *isfiilato ayaa ku dhacday* (he/she became nervous); wey waalatay/waashay (He or she gone mad), see [22] for more detailed information.

Using the above-described local Somali ethnographic perspectives, we carried out a number of forward and backward translations on the MINI. We paid particular attention to the Somali models of mental illness, which is typically expressed in terms of physical appearance and bodily complaints [22]. We discussed and resolved linguistically and culturally problematic items or items that could not be translated into Somali. After long consensus meetings and several drafts, a final Somali version of the MINI questionnaire was adopted [9]. All variables were then entered and coded using the Statistical Package for Social Sciences (SPSS 10). Initially, we carried out descriptive analyses of demographic, socioeconomic and educational data and immigration status of the research respondents. Descriptive analyses were also employed to establish the prevalence of mental health problems among this group, and Pearson’s Chi-squared tests (*χ*2) were used to examine whether a statistically significant difference existed between any two key variables, for example, between the London and Minneapolis respondents. We carried out further logistic regression modelling to examine social and contextual factors that might be associated with depression and aggregated mental disorders (ICD-10 and DSM-IV compatible), while controlling for explanatory variables such as age, sex and marital status.

## Qualitative results & discussion

In this section, the qualitative findings will be grouped under key thematic categories. These are (1) political discourses on immigration, thwarted aspirations and psychological distress; (2) unmet expectations, material conditions, changing gender roles and poor mental health and (3) pre-migration social status, loss of homeland and mental illness.

### Domestic political discourse about immigration, thwarted aspirations and psychological distress

“First of all we are refugees and being a refugee has its own disadvantage. Secondly we are black people” (lay, male, London).

“I think these two guys have said it all. You know, we are refugees and as my friend said we just do whatever we are told to do and there is nothing we can do about it” (lay, male, London).

“They don’t know that some of these people used to own big houses and cars. So they think you are a refugee who had no previous life” (lay, female, London).

As the above quotations indicate, lay participants from London felt that their new identity as refugees devalued their past lives and ruptured their personal narratives and meanings about their lives. The London participants particularly saw the attitudes of some of public service providers towards refugees as disrespectful, dehumanising and disempowering. The quote: “we just do whatever we are told to do and there is nothing we can do about it” expresses a feeling of resignation and powerlessness.

For these participants, a disparaged refugee identity was despised since this new identity brought with it a social stigma and disadvantages that affect their lives and those of their children:

“My younger brother had an argument with his teacher when he called him to a rubbish bin area and said ‘you are a refugee, aren’t you?’ and then he told him you are like this rubbish bin, you are nothing. I felt bad when he told me about this. And he is only 12 years old” (lay, female, London).

“The child will now believe that he is a refugee and he is like that rubbish bin” (lay, female, London).

Like their London counterparts, the Minneapolis lay group also noted the negative status attached to the refugee identity. Both Minneapolis and London groups made passing references to a perceived link between a devalued refugee identity and psychological distress:

“I knew this guy, he was a normal guy, he was working and he knew what was going on around him. So these illnesses are related to the stressful situations of being refugees” (lay, female, Minneapolis).

“People had different backgrounds before they came here… Of course, they will talk to themselves since they are nothing here” (professional, male, Minneapolis).

### Unmet expectations, material conditions, changing gender roles and poor mental health

Professional participants from both study sites put focus on the problems some Somalis face when they arrive in the host environment. They were particularly troubled by the complacent attitudes among the newcomers during their early days in the host nations:

“People come here and they wonder about you, so you don’t have a car, you don’t have a house, what is this, and how many years have you been here? What have you been doing in all those years? Well, I have been working all my life. The mentality when they come here is amazing. Well this is America, clean streets, tall buildings, beautiful America, people think when they are coming here they are coming to heaven only if they knew how the tall buildings were built” (professional, female, Minneapolis).

“Yeah, they would know all about that soon” (professional, male, Minneapolis).

“A young man who was told the wrong things about life in Europe and then comes to London and is expecting to have what he saw in the films. He is expecting to have a nice big house and the same bed and everything he dreamed of, he would not cope when he finds out the real life in London”, (professional, female, Minneapolis).

From the above quotations, the professional groups seem to propose that some Somalis find it difficult to integrate or overcome the initial cultural shocks they have experienced. This is partly because of the marked distance between the high living standards some Somali refugees had expected in the host nations and the actual reality of the difficult life situations in which they often find themselves, and partly because of a loss of social status compared with life in Somalia where they held professional and senior posts. This was compounded by the stigma of being a refugee and having to take on a deeply despairing new refugee identity.

“They have no good life here. They are on low wages, they experience health disparities, they take no vacation, and they go on and on until they drop dead (professional, male, Minneapolis).

The professional participants proposed that it was during this early stage when these refugees were vulnerable to breakdown under stress, partly because of the mismatch between pre-migration life expectations and status and post-migration realities of refugee existence and status. An area of crucial disagreement was the mental health consequences of unmet expectations due to changes in Somali traditional gender roles. Male professional participants stated that by coming to the Western countries, Somali men had lost their social status and that Somali women gained from this loss. This, according to the male professional, has contributed to the men’s mental health instability in the host nations:

“The Somali woman’s role is still there - to look after the children, she is studying, she is doing evening work and her roles have increased. The man had position in society, coming here, he was told you are nobody. I think that rather cracks up men. My position is not there, so that affects them”, (professional, male, London).

“I think that’s also something to do with our country, we just say a man has to have high expectations and a machismo kind of a character. But then their expectations have been dashed, whereas women, they have had low expectations initially and they're just trying to cope with their new lives. And I think that is because men go through a difficult pressure to do better whereas women who do not go through that pressure, they cope well”, (professional, female, London).

Some participants disagreed with this view stating that reverse gender roles is also bad for the psychological wellbeing of some women:

“Women with three or more unaccompanied children, can’t speak good English…She is not mixing with the community, all the time she is cooking, she can’t fill in the forms, and they are sending her more letters. That woman is all the time in need. I think that woman would go mad” (professional, male, London).

“The authority within the family has been stripped. Yes, men feel very powerless because roles have been reversed and a mother who never worked in her life when she starts to go to work here, she has to be the breadwinner in the family. She has to a lot of things to do she never used to do before but that woman is almost as vulnerable as her husband” (professional, female, Minneapolis).

Thus, changing gender roles were amongst the factors that were seen to take away prestige and dignity from Somali men, perhaps undermining their self-perceptions of their masculinity as a positive identity, with dire consequences for their self-esteem and thus mental well-being. Adverse gender roles were also seen to affect women’s mental wellbeing, although there was some disagreement as to whether men or women were more affected by the reverse gender roles in the host nations.

### Pre-migration social status, loss of homeland and mental illness

There was also a broad consensus across all groups that the collapse of the Somali state, the loss of ‘homeland’ and the ongoing conflict in Somalia has affected the psychological wellbeing of Somali refugees in the host nations:

“In my view it is the lack of government back home that is causing people to go mad. If Somalia was peaceful we wouldn’t have these problems” (lay, male, Minneapolis).

“The civil war has affected all aspects of Somali life. You can’t say that the war affected only one thing” (female, professional, London).

“That shows that the war messed up our lives” (lay, female, Minneapolis).

Participants were not just discussing the adverse psychological consequences of displacement, nostalgia and disorientation associated with the loss of a place [24], native country, but they also discussed how the current political and economic affairs taking place in the host nation are damaging to their psychological wellbeing:

“People are experiencing all sorts of new problems that they were not used to dealing with back home. These problems are what causing them too much “*Fakar*”- [thinking]” (professional, male, Minneapolis).

Lay participants were more likely than the professional participants to attribute some of the psychological problems of Somali refugees to the pressures of sending Hawala money:

“Some people are working around 18 h or 20 h a day and they still get no real benefit from their fat cheques. By the time they paid for everyone else, including relatives back home, nothing is left for them to enjoy” (professional, male, Minneapolis).

“Of course, this would have a lot of effects on people, mentally, physically and emotionally, you name it” (lay female, Minneapolis).

“It is not secret that some people do long shifts. It is not secret that some people would sleep in the factories if they were allowed. They say we would sleep inside and work for a 28 h shift”, (professional, male, Minneapolis).

According to these professional participants, many Somali refugees were under huge pressure to work on a long shift to support their extended family members. While the financial benefits of long working shifts was seen as essential for adequate survival, it was also perceived as a contributing cause of mental illness and social problems such as marriage breakdowns.

The above lay narratives all point to a collective degree of uncertainty and psychological distress caused by the lack of effective government and civil unrest in Somalia. This was a common theme across all groups. The majority of the lay and professional participants from both cities felt that Somalis had lost everything they had before the civil war including their ‘homeland’ and that this is causing psychological mal-adaptation among some Somali refugees.

In short, professional and lay Somalis from Minneapolis and London linked various psychological problems with a host of perceived pre and post-migrations risk factors. These were interconnected and included the loss of ‘homeland’, stigmatised refugee identity, unmet expectations and difficulties in the process of settlement in the host nations. Specifically, poor socioeconomic conditions and loss of former social and professional status, changes in gender roles, challenges to masculinity and thwarted aspirations, were all seen as related to psychological distress.

The similar narratives of the London and Minneapolis participants were not surprising. Both groups share a history of displacement and difficult life experiences. Discussions of clan politics (*Fadhi ku dirir*) and constant worries about the violent situation in Somalia are commonly held at Somali malls, cafés, restaurants, shops and calling centres in London and Minneapolis. The loss of a beloved ‘homeland’, the pressure to send remittance money (*Hawala*) and Somali’s current political turmoil were all narrated as a major source of psychological distress.

## Survey results

Table 3 shows differences between London and Minneapolis samples in psychological problems, demographic and immigration factors (including immigration experience and host nation integration. Of the 189 respondents, 52% were men and 48% were women. There were no statistically significant differences in age, gender, marital status, religious beliefs or area of origin in Somalia between the London and Minneapolis samples.

**Table 3:** Demographic and socio-economic characteristics and prevalence of mental disorders

**Variable**	**Value**	**London % (n)**	**Minneapolis % (n)**	***χ*2**	**df**	**P value**
Age	18-25	27.3 (39)	17.4 (8)	3.01	3	0.4
	26-35	40.6 (58)	45.7 (21)			
	36-45	21.7 (31)	19.6 (9)			
	46+	10.5 (15)	17.4 (8)			
Gender	Male	49.7 (71)	60.9 (28)	1.75	1	0.2
	Female	50.3 (72)	39.1 (18)			
Marital status	Married	50.3 (72)	60.9 (28)	1.95	2	0.4
	Never Married	42.0 (60)	30.4 (14)			
	Others	7.7 (11)	8.4 (4)			
Religion	Islam	97.9 (140)	97.8 (45)	0.001	1	0.9
	Others	2.1 (3)	2.1 (1)			
Area of origin	Rural	6.3 (9)	10.9 (5)	1.063	1	0.3
	Urban	93.7 (134)	89.1 (41)			
Mother tongue	Somali	100 (143)	100 (46)	N/A	N/A	N/A
Education in UK & US	None	33.6 (48)	58.7 (27)	9.18	1	0.002
	College/others	66.5 (95)	41.3 (19)			
Qualifications	No qualifications	51.7 (74)	60.9 (28)	1.16	1	0.280
	Certificates/Diploma	48.3 (69)	39.1 (18)			
Employment	Not working	89.5 (128)	26.1 (12)	72.9	1	0.000
	Working	10.5 (15)	73.9 (34)			
Type of work	Skilled	8.4 (12)	67.4 (31)	73.17	2	0.000
	Not skilled	2.8 (4)	6.5 (3)			
	N/A	88.8 (127	26.1 (12)			
Legal status	Pending	17.5 (25)	97.8 (45)	6.87	1	0.009
	Resolved	82.5 (118)	2.2 (1)			
Seeking asylum on entry	No	25.5 (35)	97.8 (45)	76.70	1	0.000
	Yes	75.5 (108)	2.2 (1)			
Family separation	No	31.3 (41)	56.5 (26)	9.20	1	0.002
	Yes	68.7 (90)	43.5 (20)			
Reasons for leaving home country	War	73.0 (103)	76.1 (35)	0.165	1	0.68
	Other reasons	27.0 (38)	23.9 (11)			
Length of stay in UK/US	Less than 3 years	66.4 (95)	30.4 (14)	18.47	1	0.00
	More than 3 years	33.6 (48)	69.6 (32)			
Major Depression Current	No	73.4 (105)	93.5 (43	8.23	1	0.004
	Yes	26.6 (38)	6.5 (3)			
Posttraumatic stress disorder	No	86.0 (123)	95.7 (44)	3.14	1	0.07
	Yes	14.0 (20)	4.3 (2)			
Current agoraphobia	No	89.5 (128)	100 (46)	5.24	1	0.022
	Yes	10.5 (15)				
Suicide risk	None	92.3 (132)	100 (46)	3.76	1	0.053
	Low-moderate	7.7 (11)				
Aggregated Mental Disorders	No	64.3 (92)	87 (40)	8.45	1	0.004
	Yes	35.7 (51)	13 (6)			

There were statistically significant differences of employment status between the respondents from the two cities (*χ*2 = 73.17, P ≤ .000). See Table 3. In London, 90% of the respondents were unemployed compared with 26% in Minneapolis. Compared with the London respondents, a higher proportion of employed respondents in Minneapolis were doing manual jobs (67% vs. 8%, *χ*2 = 73.2, P ≤ 0.000). Manual workers included people who were working in factories, retail shops and as taxi or lorry drivers, or doing jobs which do not require university degree qualifications. Table 3 also shows there were significant differences between the London and Minneapolis groups in terms of their immigration experience. More respondents from Minneapolis than London had obtained refugee status or citizenship (98% vs. 83%; *χ*2 = 6.87; P ≤ 0.009). There was no difference between the London and Minneapolis respondents concerning the reasons for leaving Somalia. Most respondents in both cities (73%) had fled Somalia because of the civil war (see Table 3). A greater proportion of respondents in the London group had separated from family members than in the Minneapolis group (69% vs. 44%; *χ*2 = 9.20, P ≤ 0.002). More of the London respondents had attended higher education than the Minneapolis respondents (67% vs. 41%; *χ*2 = 13.30; P ≤ .004); there were no significant differences in terms of qualifications between the London and Minneapolis respondents.

### Psychological problems

The prevalence rates of current major depression, suicide ideation and current agoraphobia were higher among the London respondents compared with the Minneapolis respondents (27% vs. 7%, *χ*2 = 8.23, P ≤ 0.004; 8% vs. 11%, *χ*2 = 5.24, P ≤ 0.022); and 11% vs. 0%, *χ*2 = 5.24, P ≤ 0.022). There were no other statistically significant differences between the London and Minneapolis respondents in terms of any single diagnostic group for mental disorders, including PTSD (See Table 3), although, overall, respondents from London showed a higher prevalence of mental disorder as is shown by the difference in aggregated mental disorders (35% vs.13%, *χ*2 = 8.45, P ≤ 0.004).

### Predictors of psychological problems

We conducted further regression analysis to examine whether the difference in the prevalence of mental disorders of the two samples can be explained by immigration differences between the respondents across the two cities. Table 4 shows the odds of major depression and aggregated mental disorders by city, after family separation, employment status and immigration factors are included at successive steps. The odds of major depression and aggregated psychological disorders among the London sample were attenuated and no longer statistically significant when we controlled for whether the respondents were employed (OR = 2.419, 0.70-8.34, 0.162; OR = 2.372, 0.51-10.95, 0.269, respectively) and for length of stay (OR = 2.562, 0.73- 8.89, 0.142; OR = 2.278, 0.48-10.71, 0.297). The odd ratio between location and psychological disorders were also reduced with the addition of family separation (OR = 4.690, 1.64-13.38, 0.004 for aggregated mental disorders; OR = 5.310, 1.36-20.63, 0.016 for major depression) and lack of legal status (OR = 4.407, 1.49-12.95, 0.007 for aggregated mental disorders; OR = 4.673, 1.16-18.75, 0.030 for major depression).

**Table 4:** Stepped model adding key explanatory variables in a model predicting the association between location, aggregated mental disorders and depression

**Variables**	**Aggregated mental disorders OR (95%CI) P**	**Major depression OR (95%CI) P**
**London/Minneapolis**	3.696 (1.46-9.30) 0.006	5.187 (1.52-17.70) 0.009
Sex and age*	4.158 (1.61-10.69) 0.003	5.951 (1.71-20.70) 0.005
Marital Status**	4.187 (1.61-10.83) 0.003	6.153 (1.74-21.69) 0.005
No Education ***	5.423 (2.01-14.60) 0.001	7.764 (2.13-28.21) 0.002
Family Separation****	4.690 (1.64-13.38) 0.004	5.310 (1.36-20.63) 0.016
Lack of legal Status*****	4.407 (1.49-12.95) 0.007	4.673 (1.16-18.75) 0.030
Unemployment ******	2.419 (0.70- 8.34) 0.162	2.372 (0.51-10.95) 0.269
Period of stay*******	2.562 (0.73- 8.89) 0.142	2.278 (0.48-10.71) 0.297

*Adjusted for sex and age +.

** marital Status +.

***Education +.

**** Family separation +.

*****Legal Status +.

****** Employment +.

*******Period of stay.

## Discussion

We used a mixed-methods approach to investigate how the social and environmental conditions in which two groups of Somali refugees live help or hinder their psychological wellbeing, and the contrasts in these conditions in different countries. The findings suggest that our samples of Somali refugees living in the two cities had similar pre-migration demographic experiences. Almost all of them were Muslim and spoke a common Somali language. The majority of them also arrived in the host nations as a result of the civil war and had experienced similar traumatic events in the country of origin, and new social adversities in the host countries.

The discussion groups explored the perceived determinants of pre and post-migration psychological distress. Some participants felt that Somalis are particularly disadvantaged in the host nations, as they are not only refugees and asylum seekers but also Muslim and Black, and often labelled as particularly hard to integrate. The narratives and experiences of some of the participants suggested that the integration experiences of Somali refugees do not easily fit into existing acculturation models [25], because of the focus of these models on the individual’s identification with the host culture, as opposed to the social and legal barriers which limit the options available for a successful integration [26]. These multiple barriers may reflect national policies, political and social attitudes to refugees and asylum seekers, entitlements to benefits and employment, and the ability to establish ties to communities and places - to belong.

The perceived stigma attached to an existence as a ‘refugee’ contributed to the sense of disillusionment and powerlessness. The narratives of the participants suggest that it was not only the unemployed or those with language problems and limited skills who were finding it difficult to integrate into the host nations, but also participants with professional skills who were fluent in English and were in a better situation to take control of their lives. They too felt that lack of recognition of their existence and previous professional skills and talents prevented them from achieving satisfactory integration, with associated risks of psychological distress.

Both the quantitative and qualitative results supported previous national studies which identified the relationship between factors in the post-migration environment and psychological well-being. There were differences between the London and Minneapolis samples; after adjustment for sex, age and marital status, Somali refugees living in London were more likely to report major depression and any mental disorder. Compared with respondents from Minneapolis, Somalis living in London experienced more problems of family separation, legal uncertainties and were more likely to be unemployed than Minneapolis respondents. These seemed to be important differences, and predictors for different risks of mental disorders in the two cities, with employment status having most of the impact by reducing the odds of major depression by a significant amount.

The quantitative study has a number of limitations. These include small samples and single cities and therefore are not necessarily representative of all Somali immigrants living in the UK and USA. On the other hand, the results are of interest because of the lack of comparative information specific to Somali immigrants and because the data were collected in a consistent way in two different international settings, allowing some unique comparisons. Although these findings cannot be generalized, consistency in the research methods used and similarities between the London and Minneapolis samples in terms of demographic characteristics made it possible to discern differences in mental health outcomes in the two samples, and to examine these differences in association with other aspects of migration experiences.

The copious qualitative data we collected also strengthened the findings of the quantitative study and therefore compensated for the limitations of the statistical method (e.g. single city samples and cross-sectional rather than longitudinal design). The professional focus groups and the Minneapolis lay discussion participants combined men and women and this may have influenced the content. For example, we do not know if the narratives of the combined male and female discussion groups would have been different from those of the single gender focus groups; and how, if any, this might have influenced the qualitative results. Despite these limitations, this study draws attention to the potential for international comparison of similar migrant groups in different host country settings, which could provide a means to evaluate the effect of variations between national policies and services for refugee populations in different countries.

## Conclusion

Although employment is a key determinant of psychological well-being for refugees, it is also dependent upon several other factors such as recognised legal status, access to language education and validation of previous professional qualifications. These risk factors have multiple effects across the different systems proposed by Silove et al’s model [7]. For example, unresolved legal status and previous tragic life events and poor psychological functioning may affect the identity/role system through a loss of status; the safety system because of restrictive state regulations, or limited employment; and the attachment-bonding system through the effects of stigma, degrading and often hostile political discourse on immigration and social isolation.

This understanding of the multiple effects of different risk factors suggests that adaptation and integration can best be achieved through policies which seek to enhance the safety, support and positive identity of refugees and which seek to reduce threats to these factors. Particularly, policy makers, social and medical practitioners are urged to consider the impact of complex needs, unemployment status, histories of adverse life trajectories, impacts of restrictive immigration policies and poor social and psychological functioning on the integration process of refugee populations. Finally, addressing the social and psychological problems of refugees would require some recognition of the ways in which the continuing conflict and social unrest in their native country are influencing their integration processes, as well negative attitudes and the difficult acculturation experiences they face after they have arrived and settled in the host countries.

## Competing interests

This project received financial support received from the UK Department of Health and from the Economic and Social Research Council (ESRC).

## Authors’ contribution

This SOMMER was funded by London Region NHS R&D Project reference no: RCC01924; Prof Bhui was the PI. NW drafted the first paper and KB, CW, KC, DI and SC have all contributed to consecutive drafts. All authors have read and approved the paper.

## Pre-publication history

The pre-publication history for this paper can be accessed here:

http://www.biomedcentral.com/1471-2458/12/749/prepub
